# Gastrointestinal Manifestations in COVID-19 Infection and Its Practical Applications

**DOI:** 10.7759/cureus.8750

**Published:** 2020-06-21

**Authors:** Amrendra Mandal, Venu Madhav Konala, Sreedhar Adapa, Srikanth Naramala, Vijay Gayam

**Affiliations:** 1 Internal Medicine, Interfaith Medical Center, Brooklyn, USA; 2 Hematology and Oncology, Ashland Bellefonte Cancer Center, Ashland, USA; 3 Hematology and Oncology, King's Daughters Medical Center, Ashland, USA; 4 Nephrology, Kaweah Delta Medical Center, Visalia, USA; 5 Rheumatology, Adventist Medical Center, Hanford, USA

**Keywords:** covid-19, ace2, gastrointestinal manifestations, pre-existing gi diseases, gi societies

## Abstract

The latest novel coronavirus (COVID-19) outbreak, which emerged in December 2019 in Wuhan, Hubei, China, is a significant cause of the pandemic. This outbreak is caused by severe acute respiratory syndrome coronavirus 2 (SARS-CoV-2) and is also commonly known as COVID-19. A typical symptom includes cough and fever, but a considerable number of patients can manifest gastrointestinal (GI) symptoms, including diarrhea, which can be the initial presentations and may or may not present with respiratory symptoms or fever. COVID-19 virus may be present in stool samples of patients infected with COVID-19, and angiotensin-converting enzyme 2 (ACE2) is a receptor for this virus, which is substantially present in GI epithelial cells. The wide availability of this receptor facilitates COVID-19 infection to be proactive and multiply in the GI tract. Although no antiviral treatments have been approved, several approaches have been proposed, and at present, optimized supportive care remains the mainstay of therapy. Elective endoscopic procedures should be delayed, but the urgent procedures should be performed as indicated. Due to the rapidly evolving data on COVID-19, it is difficult to keep up with the outpouring of information. We reviewed the mechanisms, clinical manifestation, impact on pre-existing liver diseases, and recommendations endorsed by the several GI societies for the management and prevention of its transmission.

## Introduction and background

Unexpected pneumonia was observed in early December 2019 in Wuhan, China [1]. Subsequently, a newly emerged coronavirus was labeled as SARS-CoV-2 (severe acute respiratory syndrome coronavirus 2) by the Coronavirus Study Group of the International Committee on Taxonomy of Viruses [2-4]. Coronavirus (COVID-19) resulted in a significant pandemic, with the incubation period to be 6.4 days (average), ranging from 2.1-11.1 days [5]. A wide variety of symptomology and radiographic appearances create difficulty for clinicians to identify COVID-19 and differentiate it from other more frequent respiratory infections. The usual symptoms include fever, cough, difficulty in breathing, and myalgia or fatigue, but atypically isolated sudden onset anosmia as well as a loss of taste in COVID-19 infection has also been reported [6-8]. It has also been shown that COVID-19 persists on inanimate surfaces for up to nine days; thus, it can be infective without close contact with an infected person [9]. Interestingly, patients with COVID-19 infection also demonstrated GI manifestations with diarrhea, vomiting, and abdominal pain [10]. Studies have also recognized the presence of COVID-19 virus in anorectal swabs as well as stool samples despite the virus being disappeared in the upper respiratory tract samples [11-13].

The studies demonstrated that the COVID-19 receptor, i.e., angiotensin-converting enzyme 2 (ACE2), is observed to be also expressed in gastrointestinal (GI) epithelial cells [14]. At the same time, these suggest that COVID-19 can be proactive and can significantly multiply in the GI tract. Although no antiviral treatments have been approved so far, several approaches have been proposed. This article aims to review the mechanisms, clinical manifestation, impact on pre-existing digestive diseases, and recommendations endorsed by the several GI societies for the management and prevention of its transmission. We also examine how it could impact gastroenterologists, hepatologists, transplant surgeons, and review the recommendations from different societies to mitigate the infection during the procedures.

Mechanisms of gastrointestinal tract involvement

Although COVID-19 is transmitted through respiratory droplets and close contact, indirect contact by contaminated inanimate objects also takes part in virus transmission to some extent [15,16]. ACE2 has been shown in the previous study as a receptor for different coronaviruses such as SARS‐CoV. It has also been shown that COVID-19 uses ACE2 as a viral receptor for the entry process [17]. There are existing data than ACE2 is known to be rich in the epithelial cells of the lungs and GI tracts in humans, which might facilitate the evidence of this possible route for COVID‐19 infection [18]. It is highly expressed in the glandular cells of gastric, duodenal, and rectal epithelia, supporting the entry of COVID‐19 into the host cells. In a study that analyzed COVID-19 patients, 39 (53.4%) were found to be positive for COVID‐19 in stool, with a duration of positive stool ranging between 1 and 12 days [14]. Interestingly, 17 (23.3%) patients were persistently positive for COVID-19 infection in stool even after a negative polymerase chain reaction (PCR) test in their respiratory specimens. In another study that followed 10 pediatric patients and evaluated their nasopharyngeal and rectal swabs, 8 children were persistently tested positive on rectal swabs even after nasopharyngeal clearance of the virus [19]. Zhang et al. described the presence of viral RNA in the anal swabs and fecal specimens of patients with COVID‐19 [11]. Therefore, there is evidence of fecal-oral transmission in COVID‐19 infection, and therefore rectal swab may also play a role in establishing a viral clearance.

Furthermore, a cohort study demonstrated a significant enhancement of ACE2 expression in cholangiocytes (59.7% of cells) as compared with liver cells (2.6% of cells), indicating that COVID-19 can lead to direct damage of intrahepatic bile ducts. However, the histopathological study of liver tissue from COVID-19 patients did not show viral inclusions in the liver specimens [20]. There may be other possibilities of liver abnormality in COVID-19 patients, such as drug used in the treatment itself or systemic inflammatory response induced by cases of pneumonia [10].

## Review

Liver abnormalities

The recent literature suggests that there may be mild-to-moderate injury to the liver, with elevation in aminotransferases, decline in albumin levels, and rise in prothrombin time in a patient infected with COVID-19, whereas up to 60% of patients previously infected with SARS-CoV had liver impairment [21]. The transaminases, such as aspartate aminotransferase (AST) and alanine transaminase (ALT), are elevated more than total bilirubin in the range of 14-53% in hospitalized COVID-19 patients [8]. Low serum albumin is the marker of disease severity. In one study, gamma-glutamyl transferase was elevated in 30 (54%) and elevated alkaline phosphatase levels in 1 (1·8%) of 56 patients with COVID-19 during hospitalization [22]. The histopathological examination revealed microvesicular steatosis and mild lobular activity in COVID-19 infection [20].

Gastrointestinal manifestation

Patients who presented with GI symptoms usually have a longer time from the onset of symptoms to hospital admissions than patients without these symptoms (9.0 days vs. 7.3 days). Early symptoms in the majority of COVID-19 patients have fever, myalgia, cough, and sore throat, as manifested with the other acute respiratory virus infections [23,24]. The majority of patients with COVID-19 infections present with mild symptoms, and most admitted patients have pneumonia with ground-glass opacities on chest imaging. Diagnosis becomes even more difficult considering the probability of a large number of mild or asymptomatic patients who are not recognized as a COVID-19 infection [19,25]. In one initial retrospective study from Wuhan, GI symptoms, such as diarrhea (2%‐10.1%), nausea, and vomiting (1%‐3.6%), were not very common [23,26]. However, with evolving studies related to COVID-19, up to 48.5% (204 patients) reported having GI symptoms at presentation in China. They presented with symptoms such as anorexia, diarrhea, vomiting, and abdominal pain [27].

Patients with underlying digestive diseases

COVID-19 infection may present with a severe infection in chronic liver diseases such as viral hepatitis B or C [28].

Hepatitis B virus

In the study of 1,099 patients, 23 (2.1%) patients had hepatitis B virus (HBV) infection, and severe cases had HBV infection (2.4% vs. 0.6%) than non-severe cases [28]. Currently, there are no available data on the pre-existing chronic liver disease, such as non-alcoholic fatty liver disease and alcohol-related liver disease, and their impact on the outcome of COVID-19. It is also not known if COVID-19 infection aggravates cholestasis in those with pre-existing cholestatic liver diseases such as primary biliary cholangitis or primary sclerosing cholangitis or with underlying cirrhosis [22].

Decompensated cirrhosis

There is little information about the impact of COVID-19 infection in patients with chronic liver disease. Patients with chronic liver disease should be considered for the evaluation of COVID-19 if manifested with hepatic encephalopathy, and patients with hepatic hydrothorax, portopulmonary hypertension, or hepatopulmonary syndrome also need to be considered for aggressive airway management [28].

GI tumors including hepatocellular carcinoma

There are no data on whether hepatocellular carcinoma (HCC) causes severe COVID-19 infection. However, one case series revealed worse COVID-19 outcomes with non-hepatic tumors [29]. Whether patients with other GI cancers are more susceptible to COVID-19 infection than those with non-GI cancers also remains unknown [30]. The American Association for the Study of Liver Diseases (AASLD) recommends continuing the usual HCC surveillance imaging in patients with HCC. A delay of roughly two months may be reasonable for surveillance, as the possible duration of the pandemic cannot be estimated at this time. However, if indicated, HCC treatment is recommended to initiate without further delay due to pandemic [30].

Liver transplantation

At present, there is no exact information on COVID-19 infection and its effects on decompensated cirrhosis or those in the waiting list for liver transplantation (LT). Almost all Organ Procurement Organizations (OPOs) are currently investigating for COVID-19 and will begin with negative donors; although, the capacity to investigate recipients before the proceeding with transplant may be limited. At present, there are no clear data on the impact of COVID-19 infection on patients with decompensated cirrhosis or those already on the waiting list for the LT. So far, there is no report of COVID-19 infection in transplant recipients [31]. Nevertheless, the recipient might get transmission of COVID-19 infection from a donor during LT, and, thus, donor screening is vital, as demonstrated in the earlier SARS-CoV outbreak [32]. LT surgeons are recommended to follow guidelines provided by the Transplantation Society and also follow the local health department guidelines.

Post-transplant

The evolving studies related to COVID-19 infection show that the innate immune response may be the primary driver for lung injury due to COVID-19, and, in fact, immunosuppression may be protective [33,34]. However, patients on immunosuppressive agents may have persistent viral detection in post-transplant status with COVID-19 infection and may involve in the community spread as well; thus, these patients need to be monitored to prevent the spread of infection along with monitoring of symptoms [35]. The current guidelines recommend not to decrease immunosuppression and also recommend against stopping mycophenolate for stabilized post-transplant patients with COVID-19 infection [35].

Inflammatory bowel disease

There are no data to suggest that the patients on biologics and immunosuppressive drugs in patients with inflammatory bowel disease (IBD) are more prone to COVID-19 infection. International organizations such as Crohn’s and Colitis Foundation and the International Organization for the Study of Inflammatory Bowel Disease (IOIBD) have issued helpful resources due to rising in COVID-19 infection as it relates to IBD medications [36]. The recommendation is to encourage the patients to stay on their IBD medications such as aminosalicylates. However, reducing the dose of steroids is suggested by IOIBD with a dose of 10 mg or above once daily to avoid adrenal insufficiency. Immunomodulators such thiopurines (azathioprine, 6-mercaptopurine, cyclosporine, methotrexate) and tofacitinib (JAK inhibitor) inhibit the immune response to viral infections, and the patients should not stop taking these medications. Biologics and biosimilars, including certolizumab pegol, adalimumab, infliximab, and golimumab, are immune-suppressing drugs, and patients are encouraged to continue taking these medications [36].

Treatment

Appropriate management strategies for patients with COVID-19 infections are rapidly evolving with therapeutic challenges, and optimal agents to treat an infection or prevent progression to critical illness remain unclear. Pharmacological agents such as convalescent plasma from patients who have recovered from viral infections are being used for patients with COVID-19 infection as there was some reported success during SARS-CoV-1, Middle East respiratory syndrome (MERS), Ebola, and H1N1 influenza [37]. However, the safety and efficacy of recovered convalescent plasma transfusion in symptomatic COVID-19-infected patients have not been established, and, currently, no guideline exists in the USA, but this treatment is tried on a case-to-case basis. Other agents, such as remdesivir, chloroquine, and hydroxychloroquine, are showing some good results in small studies and are currently being used in the USA after the evaluation of cardiac parameters [38-40]. The other adjunctive medications, such as lopinavir/ritonavir, tocilizumab, and corticosteroids, are also being used without many benefits [41-43]. The clinical efficacy and safety for the different agents are still under research, and, at present, optimized supportive care remains the mainstay of therapy.

Practical application

The primary route of COVID-19 transmission is through aerosolized droplets, with the possibility of fecal-oral transmission [44]. Considering that COVID-19 infection can be transmitted from an asymptomatic person and also remains detectable in stool specimens even after negative viral RNA from the lung specimen, the several GI societies have come up with joint guidelines to protect the vulnerable persons in the societies, patients, and healthcare professionals [14]. More than 40% of all COVID-19 infections may be transmitted before the index case manifesting symptoms [24,45]. Moreover, patients with GI symptoms have a longer time from onset to hospitalizations. Physicians should recognize that GI symptoms, such as diarrhea, maybe a presenting feature of COVID-19, and a high index of suspicion requires earlier at-risk patients presenting with GI symptoms.

The AASLD recommended testing for HBV and hepatitis C virus (HCV) in patients with elevated liver enzymes and monitoring liver function tests if treated with remdesivir and tocilizumab [28]. Caution should be taken regarding the use of investigational medications such as remdesivir, tocilizumab, and hydroxychloroquine for COVID-19 infection in pre-existing liver diseases. HCC surveillance may be delayed roughly to two months in patients with cirrhosis, HBV, and HCV and proceeded with HCC treatment without delay if needed [28].

The joint GI societies strongly recommend considering rescheduling non-urgent endoscopic procedures as these procedures are considered aerosol-generating [46,47]. In general, all elective procedures should be delayed, such as screening and surveillance colonoscopy in asymptomatic patients, endoscopic ultrasound (EUS) for intermediate-risk pancreatic cysts, and motility procedures [47]. However, urgent/emergent procedures should not be delayed, such as GI bleeding treatment, esophageal foreign body impaction, endoscopic retrograde cholangiopancreatography (ERCP) for acute cholangitis, EUS drainage for symptomatic pancreaticobiliary disease, and palliation of GI obstruction [47].

The several COVID-19-related studies reporting about GI manifestation have been compared in Table 1.

**Table 1:** Comparisons of GI manifestation in several studies GI, gastrointestinal

Study	Sample	Nausea	Vomiting	Diarrhea	Anorexia
Guan et al. [31]	1,099	55 (5.0%)	55 (5.0%)	42 (3.8%)	
Wang et al. [26]	138	14 (10.1%)	5 (3.6%)	14 (10.1%)	
Xiao et al. [14]	73	-	-	26 (35.6%)	
Pan et al. [27]	204	-	8 (3.9%)	29 (14.2%)	83 (40.6%)
Lu et al. [48]	171	11 (6.4%)	-	15 (8.8%)	
Liu et al. [49]	137	-	-	11 (8%)	
Zhou et al. [50]	141	7 (3.7%)	7 (3.7%)	9 (4.7%)	

## Conclusions

In this review, we summarized that GI symptoms may be a presenting feature of COVID-19 infection and that a high index of suspicion requires earlier at-risk patients presenting with GI symptoms. We also summarized the guidelines from the joint GI societies for elective GI procedures as well as urgent or emergent procedures and the impact of COVID-19 infection on the several digestive diseases and their management during a pandemic situation.
